# The Utilization of Indoleacetic Acid to Enhance the Tolerance of Microalgae to Antibiotics, Removal Capability, and Lipid Production

**DOI:** 10.3390/microorganisms14040769

**Published:** 2026-03-27

**Authors:** Lifeng Wang, Yibo Zhang, Zhenbing Wu, Chenyuan Dang, Jie Fu

**Affiliations:** 1 Hubei Key Laboratory of Multi-Media Pollution Cooperative Control in Yangtze Basin, School of Environmental Science and Engineering, Huazhong University of Science and Technology, Wuhan 430074, China; lifengw080@gmail.com (L.W.); ybo_zhang@sina.com (Y.Z.); 2021509004@hust.edu.cn (Z.W.); dangcy@hust.edu.cn (C.D.); 2 Green Energy Industry Research Centre (GEIRC), Huazhong University of Science and Technology, Wuhan 430074, China; 3 Hubei Key Laboratory of Purification and Application of Plant Anti-Cancer Active Ingredients, Hubei University of Education, Wuhan 430205, China

**Keywords:** microalgae, phytohormones, antioxidant system, antibiotics removal, lipid production

## Abstract

The utilization of microalgae for bioremediation presents a highly promising and cost-effective approach, offering advantages of simultaneous pollutant removal and biomass recovery. However, pollutants may adversely affect microalgal growth, potentially compromising both pollutant removal efficiency and biomass yield. In this study, the plant hormone indoleacetic acid was employed to enhance the tolerance to pollutants and lipid production capability of *Chlorella vulgaris* (*C. vulgaris*). Compared to the non-treated group, the addition of indoleacetic acid resulted in increased biomass, pigment content, sedimentation performance, lipid productivity and content, as well as antibiotic removal capacity in *C. vulgaris*. Furthermore, the effects of indoleacetic acid on *C. vulgaris* growth were elucidated through changes in antioxidant enzymes and fatty acid saturation. Overall, this study reveals the potential of plant hormones in enhancing microalgal tolerance and lipid productivity, providing a theoretical basis for the effective utilization of microalgae in achieving simultaneous pollutant removal and biofuel production.

## 1. Introduction

Antibiotics are extensively utilized in human healthcare, aquaculture, livestock farming, and the food industry, leading to their substantial release into aquatic environments [1]. Sulfamethoxazole (SMX), a sulfonamide antibiotic, exemplifies this widespread use, with an estimated global application exceeding 84,240 tons in human and veterinary medicine [2]. Due to the limited treatment capacity of wastewater treatment plants (WWTPs) and the self-purification capability of natural water bodies, approximately 15–25% of the administered SMX is discharged into natural waters [3]. Additionally, SMX is one of the most frequently detected antibiotics in various aquatic environments [4]. In certain areas, such as coastal regions impacted by hospital and urban wastewater, SMX exhibits a 100% detection frequency [5]. Although the mean concentration of SMX has been detected at approximately 0.342 and 0.051 μg/L in WWTP effluent and surface water, respectively [6,7], an extremely high concentration of approximately 54.83 mg/L has been detected in hospital wastewater [8]. Consequently, its potential environmental risks are attracting increasing scientific and regulatory concern [9]. Generally, even trace amounts of SMX in natural ecosystems (usually 70–150 ng/L in natural aquatic environments) exert adverse effects on non-target organisms [10]. Owing to its lipophilic properties, SMX can permeate cellular membranes, induce the generation of reactive oxygen species (ROS), and disrupt cellular homeostasis. It has been shown to significantly impair critical functions in aquatic plants, including photosynthesis and growth [11].

Microalgae are capable of converting incident solar energy into chemical energy, which is the basis of the food chain, and the reduction in its population will directly affect the balance of the entire aquatic ecosystem [12]. The limitations of natural resources and energy sources have led to increased attention on microalgae with higher biomass accumulation efficiency, nutrient assimilation, and productivity [13]. Microalgae exhibit rapid growth rates, thrive in diverse environmental conditions, and possess high photosynthetic efficiency and lipid production capabilities, offering greater sustainability and commercial advantages compared to other energy sources [1]. Compared to other plant-based sources, the simplicity of microalgae cultivation enables it to be cultivated in any country worldwide [14]. Furthermore, non-soil cultivation reduces competition with food crops for land, thus benefiting food production and other agricultural products [15].

Algae play a pivotal role in environmental applications and are recommended by the Organization for Economic Co-operation and Development (OECD) as model species for chemical ecotoxicity assessment [16]. Critically, algal sensitivity to contaminants exhibits marked interspecies variability [1]; green algae often underestimate true toxicity, whereas cyanobacteria may produce an amplification effect. Notably, sensitivity to antibiotics may correlate with removal capacity, linking ecotoxicological response to bioremediation potential [17]. Recently, the microalga *C. vulgaris* has been established as a prototypical model species in studies investigating antibiotic stress responses, owing to its unequivocal sensitivity to SMX exposure [1] and well-characterized stress response mechanisms [18]. In addition, compared to another widely used algae model, *Chlamydomonas reinhardtii,* although *Chlamydomonas reinhardtii* exhibits more favorable lipid-production traits, *C. vulgaris* remains the preferred genus for biodiesel production on a mass production scale [19] due to its exceptionally high biomass productivity under optimized heterotrophic conditions [20]. Notably, the application of microalgae in environmental mitigation has advanced significantly since the elucidation of algal-bacterial mutualism in 1955 [21]. Leveraging synergistic principles such as interspecific substrate cycling, metabolite exchange, and community complementarity, this consortium demonstrates marked potential in wastewater treatment, bioremediation, and biorefining [22]. Recently, to understand and engineer algal-bacterial consortia for environmental applications, studying complex chemical dialogue among algal-bacterial mutualism and discovering genetic and mechanistic foundations have become popular. With the molecular basis of auxin-mediated interkingdom signaling elucidated by Calatrava et al. [23], genetic evidence for auxin biosynthesis in a microalga was first provided. It revealed that algal-produced IAA served as a key mediator of mutualistic interactions with plant-growth-promoting bacteria such as *Methylobacterium aquaticum*, which can degrade IAA to modulate its inhibitory effects on algal growth under nitrogen limitation. Additionally, Amin et al. [24] identified bacterial auxin as a core mediator of interspecies material exchange within such consortia. Moreover, long-term acclimated algae have been observed to augment their resistance to antibiotics and enhance their capacity for antibiotic removal, potentially resulting in the emergence of novel degradation byproducts [25]. However, relevant studies have not confirmed whether domestication can maintain long-term effectiveness.

Phytohormones, as crucial secondary metabolites secreted by algae, have been demonstrated to effectively mitigate the impacts of abiotic stresses on algal physiology (i.e., salinity and heavy metal stresses) [26]. By promoting algal lipid synthesis [27], this process may enhance the algal capacity for pollutant removal. Auxin, the first discovered class of phytohormones, plays a pivotal role in key metabolic activities, including plant growth and stress responses [28]. Indole-3-acetic acid (IAA) is recognized as the primary endogenous auxin in microalgae and is widely distributed across multiple algal phyla, including Chlorophyta, which comprises most *Chlorella* species [29]. For example, a comprehensive screening by Žižková et al. [30] confirmed the presence of endogenous IAA in a phylogenetically diverse set of twenty algal strains, encompassing members of the *Haptophyta*, *Chlorophyta*, and *Streptophyta,* etc. Subsequently, it has demonstrated that exogenous supplementation of L-tryptophan significantly enhances IAA production in various algal species, which supports the existence of a tryptophan-dependent IAA biosynthesis pathway in microalgae [31]. Currently, Calatrava et al. [23] first identified a novel IAA biosynthesis pathway in *Chlamydomonas reinhardtii* mediated by an L-amino acid oxidase (LAO1). LAO1 catalyzes the oxidative deamination of L-tryptophan to produce indole-3-pyruvate, representing a crucial initial step in IAA synthesis in this alga. However, to date, definitive molecular biological evidence for a homologous LAO1-mediated pathway in *Chlorella* species remains lacking.

Meanwhile, IAA as a synthetic auxin, has been widely used in agriculture; the concentration of IAA distributed in wastewater and sludge has been detected about 0–586 ng/L and 0.5–2.6 ng/g, respectively [32,33]. The effects of IAA on microalgae were reflected in the adjustment of various metabolic activities. Firstly, a seminal observation by Stirk et al. [27] demonstrated enhanced plant growth following the application of auxin-rich brown algal extracts. Subsequently, endogenous IAA production has been established as a widespread trait across diverse microalgae, with the IAA biosynthetic pathway confirmed in the model organism *Chlamydomonas reinhardtii* [23].

Beyond endogenous synthesis, IAA functions as a key signaling mediator in algal-bacterial interactions. Furthermore, exogenous IAA supplementation mitigates abiotic stress effects on microalgae by stimulating pigment accumulation, energy metabolism, and antioxidant responses, thereby enhancing biomass production [34,35]. However, research on the restorative effects of exogenous IAA on microalgal activity under antibiotic stress remains notably absent. Therefore, this study aims to investigate the concurrent removal of pollutants and recovery of microalgal biomass through the application of IAA.

In the present study, the plant hormone indoleacetic acid was added to the *Chlorella vulgaris* (*C. vulgaris*, FACHB-8) culture system to enhance the algae’s tolerance to antibiotics. The objectives of this work were (1) to assess the effects of indoleacetic acid on the tolerance of *C. vulgaris* to antibiotics; (2) to investigate the effects of indoleacetic acid on the mechanisms underlying antibiotic removal by *C. vulgaris*; and (3) to compare the effects of indoleacetic acid on the biomass content and composition of *C. vulgaris*.

## 2. Materials and Methods

### 2.1. Microalgae Culture and Chemicals

*C. vulgaris* was purchased from the Freshwater Algae Culture Collection at the Institute of Hydrobiology and cultured in BG11 medium. All instruments and culture media used for inoculation were sterilized in an autoclave at 105 °C for 30 min, and the culture of algae was maintained under strictly axenic conditions. The temperature in the light incubator was maintained at 25 ± 2 °C, with a light intensity of 4000 lx and a light/dark cycle of 16 h/8 h. Prior to the formal experiment, pre-cultivation was conducted for two weeks to allow acclimation to laboratory conditions.

SMX was purchased from Shanghai Yuanye Bio-Technology Co., Ltd. (Shanghai, China). Methanol and acetonitrile were obtained from Shanghai Macklin Biochemical Co., Ltd (Shanghai, China). Indoleacetic acid was purchased from Merck KGaA (Darmstadt, Germany). Other reagents were purchased from Sinopharm Chemical Reagent Co., Ltd (Shanghai, China).

### 2.2. Experimental Procedure

In this study, batch cultures of *C. vulgaris* were established by inoculating cells (during the logarithmic growth phase) into 200 mL of BG11 medium in 250 mL flasks with an initial concentration of 1 × 10^4^ cells mL^−1^, following reported procedures [36]. The study was organized into two main groups: one receiving IAA (dosage of 10 mg/L) and the other without IAA supplementation. Within two main groups, six concentration levels of SMX (0, 1, 3, 5, 7, and 9 mg L^−1^) were set to run separate batch experiments with three independent biological replicates (*n* = 3). The schematic of the experimental setup is provided in Table 1. Additionally, a stock solution of SMX (1 g/L, >99% purity) was prepared by dissolving the standard in chromatographic-grade methanol (volume ratio = 1:1000). It was set at a high concentration of SMX, with the purpose of inducing sufficiently pronounced and measurable physiological effects within a finite experimental timeframe (i.e., growth inhibition, oxidative damage, and alterations in fatty acid synthesis) [37].

The experimental period lasted for 11 days. To ensure normal growth of *C. vulgaris* and minimize experimental errors, we shook the culture bottles five times daily, with each shake followed by random repositioning of the bottles.

### 2.3. Measurement Methods

#### 2.3.1. Growth Curve and Biomass

Cell numbers were determined following the method described in previous studies [1]. Briefly, cell numbers were counted using the hemocytometer and optical microscope. Simultaneously, the absorbance (OD_680_) of the algal suspension with different cell densities was measured. The curve-fitting analysis was performed using the obtained cell numbers and OD_680_ values, and the following equation was derived:

(1) Cell numbers (cells/mL) = 3315.2 × OD_680_ − 76.4 (R^2^ = 0.994)

#### 2.3.2. Determination of Photosynthetic Pigment and Photosynthetic Activity

To extract photosynthetic pigments, a 5 mL aliquot of the algal suspension was centrifuged at 10,000 rpm for 5 min. The resulting supernatant was discarded, and the cell pellet was resuspended in 5 mL of ethanol. The resuspended mixture was then stored in an ultra-low temperature freezer at −80 °C for 24 h. Following this incubation, the sample was centrifuged again at 10,000 rpm for 5 min. The supernatant was collected, and the contents of chlorophyll a, chlorophyll b, and total carotenoids were quantified using the following formulas.

(2) Chlorophyll a (mg/L) = 15.65A_666_ − 7.34A_653_

(3) Chlorophyll b (mg/L) = 27.05A_653_ − 11.21A_666_

(4) Total carotenoids (mg/L) = (1000A_470_ − 44.76A_666_)/221

A_666_, A_653_, and A_470_ denote the absorbance of the supernatant, respectively.

Photosynthetic activity was monitored via chlorophyll fluorescence using a PAM 101–103 system (H. Walz, Effeltrich, Germany) with an ED-101US detector. Samples were diluted to 4 mg/L chlorophyll in fresh medium. Dark-adapted (10 min) 1.5 mL samples, collected from the light incubator at the end of the experiment, were analyzed for Fv/Fm. Fo was obtained under modulated 655 nm LED light (<0.3 μmol photons m^−2^ s^−1^, 1600 Hz), and Fm was induced by a 0.8 s saturating flash (5500 μmol photons m^−2^ s^−1^) from a Schott K2 1500 halogen lamp (FL-103/E.220) [38]. The calculation of chlorophyll fluorescence (Fv/Fm) was according to the following formula:

(5) Fv/Fm = (Fm − Fo)/Fm

#### 2.3.3. Enzyme Activity and MDA Content

At the end of the experiment, the algal suspension (10 mL) was centrifuged at 10,000 rpm for 10 min, and the supernatant was discarded. The pellet was washed three times with physiological saline solution. The contents of malondialdehyde (MDA), superoxide dismutase (SOD), catalase (CAT), and glutathione peroxidase (GSH-Px) were determined using assay kits purchased from Nanjing Jiancheng Bioengineering Institute, with units of U/g, and all procedures were performed according to the manufacturer’s instructions.

#### 2.3.4. Analysis of Antibiotics

Upon completion of the 11-day cultivation, the contributions of biosorption, bioaccumulation, and biodegradation to SMX were investigated across the different experimental groups. The concentration of SMX was determined using liquid chromatography tandem mass spectrometry (LC-MS/MS, Agilent 1290/6460, Santa Clara, CA, USA). The relevant parameters of the instrument can be found in the Supporting Materials. To determine the residual SMX concentrations, 30 mL aliquots of the algal suspension were collected from each group and centrifuged at 8000 rpm for 10 min. The resulting supernatant was filtered through a 0.22-μm membrane filter and used for the quantification of initial and residual SMX. The algal pellet was resuspended in 30 mL of ultrapure water and washed three times; the supernatants from these washes were collected to determine the biosorption amount of SMX. Following, the pellet was again resuspended in ultrapure water and disrupted by ultrasonic cell crushing (150 W) for 15 min, after which the homogenate was centrifuged. The supernatant obtained was subsequently used for the quantitative measurement of bioaccumulated SMX. Finally, the biodegradation amount of SMX was calculated by subtracting the amounts of residual, bioaccumulated, and biosorbed SMX from the initial SMX concentration.

#### 2.3.5. Sedimentation Ratio and Zeta Potential

0.1 mL of algal suspension, 4.65 mL of 5 g/L kaolin suspension, and 0.25 mL of 90 mM CaCl_2_ (9.9882 g dissolved in 1 L water) were uniformly mixed in a vortex mixer and shaken for 30 s, followed by a 5 min settling period. The supernatant was then collected, and its optical density (OD) at 550 nm was measured using ultraviolet-visible spectrophotometry [39]. This experiment was independently repeated three times.

(6) Sedimentation ratio (%) = (OD_500,b_ − OD_500,a_)/OD_500,b_

where OD_550,a_ and OD_550,b_ denote the absorbance of the control and the treatment groups, respectively.

Zeta potential measurements were performed on a Zetasizer Nano ZS90 instrument (Malvern, UK). Each reported value is the average of three independent measurements, and the error bars correspond to the standard deviation. The pH of the samples was approximately 7.0–7.5 and was not adjusted prior to analysis.

#### 2.3.6. Lipid Content and Fatty Acids Composition

Lipid content and lipid productivity were determined using the method described in previous studies [40]. In brief, algal cells were dissolved in chloroform and methanol (volume ratio 2:1), followed by centrifugation at 4000 rpm to collect the supernatant. The supernatant was then dried using nitrogen blowing to obtain the extraction yield and lipid productivity. A one-step extraction esterification method was used for the determination of fatty acid methyl ester, and gas chromatography-mass spectrometry (GC-MS) was used to analyze fatty acid methyl ester [41].

### 2.4. Transcriptome Sequencing and Analysis

On day 11 of experiment, microalgal samples were collected from the groups. The groups were defined as follows: CK (control), IAA (treated with indoleacetic acid only), CKSMX (treated with 5 mg/L SMX only), and IAASMX (co-treated with indoleacetic acid and 5 mg/L SMX). Three replicate samples for each group were subsequently sent to Majorbio Co., Ltd. (Shanghai, China) for transcriptome sequencing. The transcriptomic sequencing with the PE150 strategy was performed on the Illumina Nova-Seq platform (Novogene, Beijing, China); libraries were sequenced on an Illumina platform to generate paired-end reads. An average sequencing depth of 30 to 50 million clean reads per biological replicate was achieved to ensure robust gene expression quantification and differential analysis. The raw sequences were subsequently deposited in the Sequence Read Archive (SRA) under project number PRJNA1430207. The raw gene expression data were then normalized. Differential expression analysis was performed using the DESeq2 package (version 1.50.2) to compare the transcriptomic profiles between the relevant experimental and control groups. Differentially expressed genes (DEGs) were identified with a stringent threshold of an adjusted *p*-value (*p*adj) < 0.05 and an absolute log2 fold change (|log2FC|) ≥ 2. Finally, functional annotation of the identified DEGs was carried out through enrichment analyses based on the Gene Ontology (GO) and Kyoto Encyclopedia of Genes and Genomes (KEGG) databases.

### 2.5. Statistical Analysis

All experiments were conducted in triplicate, and error bars represent mean ± standard deviation. Statistical analysis was conducted using ANOVA after the determination of variances with significance set at *p* < 0.05. All figures were generated using Origin (version 2019b) and GraphPad Prism (version 10.0); the combination and modification of the figures were carried out using PowerPoint.

## 3. Results

### 3.1. The Growth, Photosynthetic Pigment Difference Between CK Group and IAA Group

**Figure 1:**
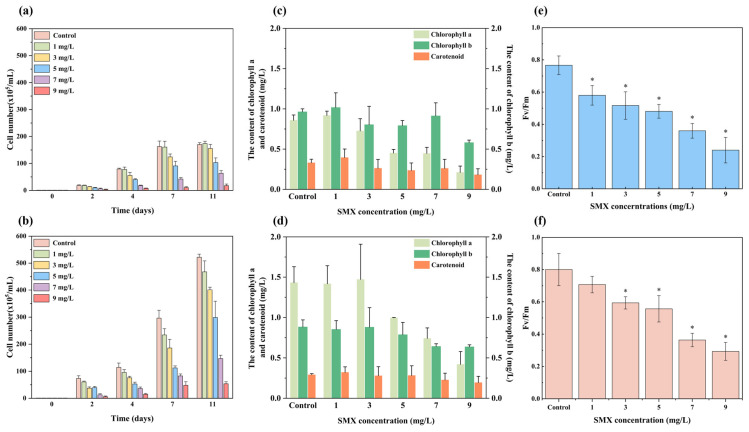
The growth curve, pigment contents, and photosynthetic activity of (**a**,**c**,**e**) the non-treated and (**b**,**d**,**f**) the IAA-treated group. Significant differences (Tukey’s post-hoc test) at *p* < 0.05 are shown with *. Error bars show the standard deviations of triplicate results.

Both the CK and IAA groups exhibited a dose-dependent growth inhibition in response to SMX stress, with IAA-supplemented cultures maintaining significantly higher cell densities than their non-treated counterparts throughout the experimental period (Figure 1a,b). After being cultured for 11 days under SMX stress, pigments and Fv/Fm of *C. vulgaris* were detected. Chlorophyll a and b contents declined progressively in both groups, reaching minima of 0.18 mg L^−1^ and 0.58 mg L^−1^, respectively, at 9 mg L^−1^ SMX (Figure 1c,d). IAA supplementation significantly mitigated this decline; at 3 mg L^−1^ SMX, chlorophyll a content in the IAA group was 2.04-fold higher than that in the SMX-only group (*p* < 0.05). The effect of both SMX and IAA was more pronounced on chlorophyll a than on chlorophyll b or carotenoids.

The Fv/Fm ratio represents the maximum photochemical efficiency of Photosystem II, reflecting the proportion of absorbed light energy that is converted to chemical energy via photochemistry. It is a sensitive and widely used indicator of photosynthetic performance and serves as an early warning signal of photoinhibition and damage to the photosynthetic apparatus under stress conditions [22]. Fv/Fm values decreased with increasing SMX concentration, with the lowest values of 0.34 and 0.47 recorded at 9 mg L^−1^ SMX for the CK and IAA groups, respectively (Figure 1e,f). Across all SMX concentrations, Fv/Fm remained consistently higher in IAA-treated cultures than in the corresponding CK groups. Despite IAA supplementation, Fv/Fm continued to exhibit a concentration-dependent decline under SMX stress.

### 3.2. The Difference of Antioxidant System Between Indoleacetic Acid Group and Non-Treated Group

**Figure 2:**
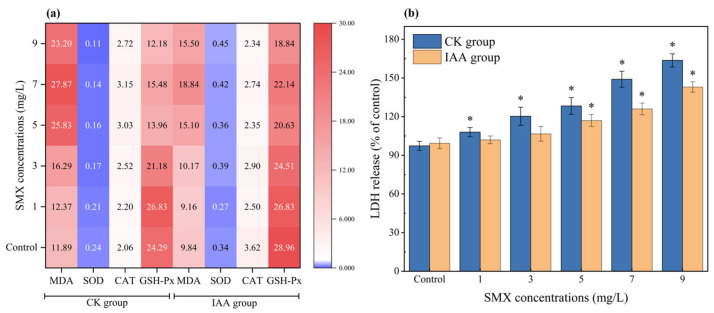
Effect of SMX on activity of antioxidant system (**a**) and LDH release (**b**) of *C. vulgaris* in non-treated groups and IAA-treated groups. Significant differences (Tukey’s post-hoc test) at *p* < 0.05 are shown with *. Error bars show the standard deviations of triplicate results.

MDA, an aldehydic byproduct of ROS-mediated lipid peroxidation, is a widely recognized biomarker for assessing contaminant-induced oxidative damage in organisms [42]. It is observed that exposure to SMX significantly increased the MDA content in *C. vulgaris* relative to the control. A distinct differential response was evident between the IAA-supplemented and non-treated groups. In the absence of IAA, MDA content increased markedly once the SMX concentration exceeded 3 mg L^−1^. By contrast, in the IAA-treated group, MDA levels remained largely stable at SMX concentrations below 7 mg L^−1^, with only minor fluctuations (Figure 2a). Meanwhile, lactate-dehydrogenase (LDH) release serves as an established indicator of cell membrane integrity, and previous studies have employed this parameter to evaluate antibiotic-induced membrane damage in microalgae [11,43]. SMX exposure induced LDH release, whereas the addition of IAA significantly reduced this release (Figure 2b).

Furthermore, as the first line of defense in the antioxidant system of microalgae, SOD and CAT primarily function to counteract oxidative stress induced by abiotic stresses through the scavenging of reactive oxygen species (ROS). Specifically, SOD catalyzes the dismutation of superoxide radicals (O_2_^−^) to hydrogen peroxide (H_2_O_2_) and molecular oxygen (O_2_), and the resulting H_2_O_2_ is subsequently converted to water (H_2_O) and O_2_ by CAT [44]. In the present study, SOD activity in the CK group declined significantly at high SMX concentrations but showed no significant decrease at low concentrations. In the IAA-treated group, SOD activity decreased slightly at low SMX concentrations (not significant) and increased significantly under high SMX stress (Figure 2a). Elevated SOD activity facilitates the scavenging of superoxide radicals, thereby preventing downstream cellular damage [1]. Meanwhile, other enzyme contents comprising the antioxidant defense system revealed similar changes. For example, high SMX concentrations provoked a pronounced increase in CAT activity in the non-treated group, while CAT activity in IAA-treated cells decreased marginally across all SMX concentrations tested (Figure 2a). Moreover, GSH-Px activity decreased in *C. vulgaris* upon SMX exposure, with the non-treated group exhibiting consistently lower activity than the IAA-treated group (Figure 2a).

### 3.3. Removal Efficiency of SMX in Different Treated Groups

**Figure 3:**
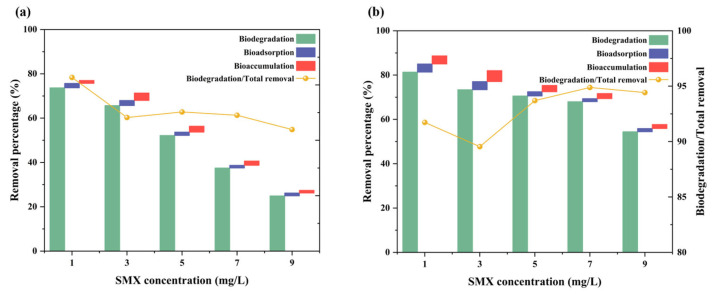
The SMX removal rate of different mechanisms by *C. vulgaris* in the CK group (**a**), and the IAA group (**b**).

In the coexistence systems of microalgae and antibiotics, the removal mechanisms for antibiotics primarily include biosorption, bioaccumulation, biodegradation, photodegradation, and volatilization. For SMX, its significant hydrophilicity and low Henry’s law constant render its volatilization from aqueous environments negligible [45]. Direct or indirect photodegradation typically requires ultraviolet (UV) radiation and the presence of photosensitive DOM components; given that the incubation conditions lacked a UV component and that SMX exhibits limited photodegradation under alkaline conditions [25,46], the contribution of photodegradation to SMX removal in this study was considered minimal and thus disregarded. In this context, biosorption of SMX was observed in groups of both with IAA and without IAA, exhibiting a higher adsorption capacity than the CK group, particularly at lower SMX concentrations (Figure 3). Notably, biodegradation constituted the predominant mechanism for SMX removal; however, its proportional contribution to overall removal decreased progressively with increasing SMX concentration. However, IAA supplementation enhanced total SMX removal efficiency, and this promoting effect on biodegradation was especially pronounced under high-concentration SMX stress. At 9 mg L^−1^ SMX, IAA increased the biodegradation efficiency by nearly 20% (Figure 3). Overall, SMX removal was primarily driven by biodegradation, with biosorption serving as a supplementary mechanism.

### 3.4. Biomass Harvesting and Lipid Content

**Figure 4:**
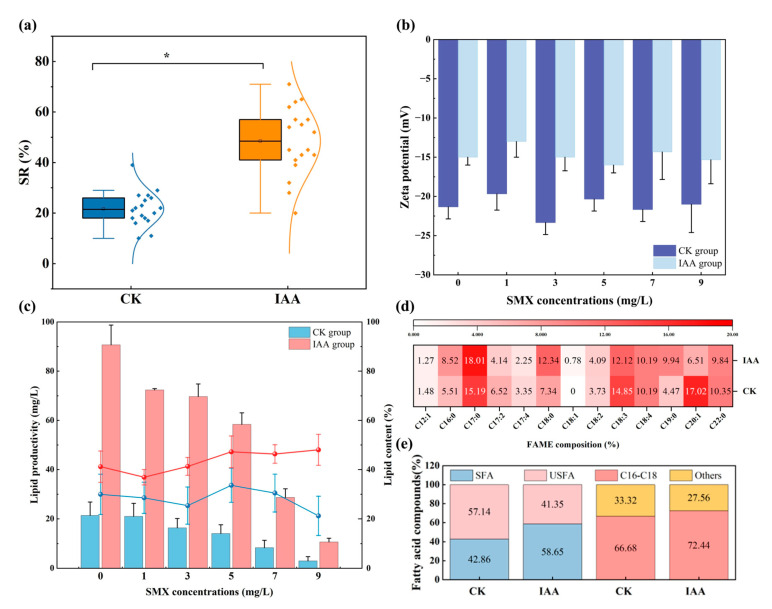
The difference between the non-treated group and the indoleacetic acid group in SR (**a**), zeta potential (**b**), lipid productivity and content (**c**), fatty acid profile (**d**), and fatty acid composition (**e**). Significant differences (Tukey’s post-hoc test) at *p* < 0.05 are shown with *. Error bars show the standard deviations of triplicate results.

In terms of improving the accumulation and acquisition of microalgae biomass, the research first demonstrated that SMX addition inhibited algal biomass growth, whereas co-supplementation with IAA significantly mitigated this inhibitory effect (Figure 1). Furthermore, IAA treatment increased the settling velocity of *C. vulgaris* by approximately 23% (*p* < 0.05), thereby enhancing biomass recovery (Figure 4a).

In parallel, the absolute zeta potential of IAA-treated cells was lower than that of the non-treated group (Figure 4b). At the end of the experiment, lipid content and lipid productivity were measured. Lipid productivity in both CK and IAA groups declined progressively with increasing SMX concentration; however, values in the IAA groups remained significantly higher than those in the corresponding CK groups (Figure 4c).

While lipid content (percentage of dry weight) was not substantially affected by SMX concentration, total lipid yield varied as a consequence of SMX-induced changes in overall biomass. As presented in Figure 4d,e, the proportion of saturated fatty acids (SFA) in the CK group was 42.86%, compared to 58.65% in the IAA group. Moreover, IAA supplementation increased the proportion of C16–C18 fatty acids in *C. vulgaris*.

### 3.5. Genes Involved in Photosynthesis, Energy Metabolism, Fatty Acid Synthesis, and SMX-Resistant Pathways

To elucidate the effects of IAA regulation on *C. vulgaris* metabolism, transcriptomic sequencing and differential gene expression analysis were performed across four experimental groups. Exogenous application of IAA significantly altered the expression of functional genes associated with key metabolic pathways. SMX stress markedly suppressed the expression of photosynthesis-related genes, whereas IAA supplementation consistently reversed this inhibition, which was consistent with the observed pigment loss and Fv/Fm decline. Under SMX stress, IAA induced substantial upregulation of photosynthetic genes. Compared to the CKSMX group, the IAASMX group exhibited a 2.45-fold increase in *psbY* (encoding a photosystem II core protein), a 6.13-fold increase in *petH* (encoding ferredoxin-NADP^+^ reductase), a 4.13-fold increase in *atpG* (encoding F-type ATPase gamma subunit), a 1.98-fold increase in *psaN* (encoding photosystem I subunit), and a 4.52-fold increase in *petN* (encoding cytochrome b_6_/f complex subunit). Moreover, both the Calvin cycle and the TCA cycle were significantly upregulated under SMX stress alone. The expression of *rbcL* (encoding the RuBisCO large subunit) increased 1.92-fold in the CKSMX group relative to the CK group, accompanied by elevated *ripA* (encoding ribose-5-phosphate isomerase). The *CS* gene (encoding citrate synthase) was upregulated 1.24-fold [34]. In contrast, IAASMX samples displayed generally lower expression levels of these genes compared to CKSMX.

SMX stress broadly downregulated fatty acid biosynthesis genes, whereas IAA co-treatment reversed this suppression and further enhanced pathway activity. Notably, *ACAC* (encoding acetyl-CoA carboxylase) was upregulated 2.31-fold in IAASMX versus CKSMX, alongside marked induction of elongation genes *fabG*, *fabI*, and *fabF*.

Genes encoding cytochrome P450 monooxygenases were moderately upregulated under SMX stress alone, but IAA supplementation elicited substantially stronger activation. Relative to CKSMX, IAASMX showed 1.69-, 1.38-, and 2.96-fold increases in *CYP55*, *CYP51*, and *CYP120A1*, respectively. Additionally, *GLU* (encoding glutamate synthase) and *DHFS* (encoding dihydrofolate synthetase), which are implicated in SMX acetylation and formylation pathways, were upregulated 1.76-fold and 1.45-fold, respectively. SMX exposure induced significant upregulation of *glnA* (encoding glutamine synthetase) and *GSS* (encoding glutathione synthetase). IAA supplementation further amplified this response, with *gshA* (encoding glutamate–cysteine ligase) expression increasing 3.36-fold in IAASMX relative to CKSMX.

## 4. Discussion

### 4.1. IAA Enhances Antibiotic Tolerance Through Dual Antioxidant and Biodegradation Mechanisms

Firstly, growth performance could serve as a direct indicator of algal tolerance to pollutant stress. The significantly higher cell densities observed in IAA-treated cultures demonstrate that exogenous IAA could effectively promote the growth of *C. vulgaris* under SMX stress, thereby enhancing its stress tolerance. Secondly, photosynthetic pigments are essential for converting solar energy into chemical energy to sustain normal growth [1]; thus, changes in pigment content are closely associated with microalgal biomass responses to hazardous substances [47]. The observed reduction in chlorophyll a and b under SMX stress may reflect a classical physiological response to contaminant exposure, typically attributed to thylakoid membrane lipid peroxidation and degradation of the photosystem II (PSII) complex [48]. The marked attenuation of this pigment loss by IAA supplementation suggests that IAA helps preserve photosynthetic integrity. The elevated chlorophyll content likely enhances the cell’s intrinsic capacity to scavenge reactive oxygen species (ROS) accumulated within chloroplasts, thereby aiding in the mitigation of SMX-induced oxidative stress. In addition, carotenoids contribute to photosynthesis by broadening the spectral range of light absorption and play a critical photoprotective role by quenching singlet oxygen and free radicals, thereby preserving membrane integrity and preventing oxidative damage to the photosynthetic apparatus under adverse conditions such as high light, temperature extremes, salinity, and pollutant exposure [25,49]. The preservation of carotenoid content by IAA under moderate SMX stress may have contributed to the sustained photosynthetic performance observed. The more pronounced effect of both SMX and IAA on chlorophyll a is consistent with prior transcriptomic evidence that SMX specifically targets genes involved in chlorophyll a biosynthesis in green algae [47], whereas chlorophyll b and carotenoid synthesis pathways were not substantially perturbed under the experimental conditions employed.

The progressive decline in Fv/Fm with increasing SMX concentration confirms that SMX impairs the algal energy conversion process. This impairment likely arises from SMX-induced disruption of photosynthetic electron transport or inhibition of PSII reaction center protein synthesis. Such effects subsequently lead to disruption of pigment synthesis (Figure 1) and energy transformation [1]. The improved Fv/Fm values in IAA-treated cultures demonstrate that IAA supplementation could mitigate SMX-induced photoinhibition and enhance photosynthetic efficiency. IAA may protect PSII function by promoting chlorophyll biosynthesis (as evidenced by increased chlorophyll a content), stabilizing the PSII complex, or upregulating antioxidant enzymes that alleviate oxidative stress [38]. These actions help maintain the integrity of the photosynthetic apparatus and facilitate more efficient light energy conversion under antibiotic stress. Furthermore, the observed changes in Fv/Fm could be mechanistically linked to the expression patterns of photosynthesis-related genes. Under SMX stress, IAA supplementation significantly upregulated key genes involved in the photosynthetic light reactions, including *psbY* (encoding a PSII core protein), *petH* (ferredoxin-NADP^+^ reductase), *atpG* (ATP synthase), *psaN* (PSI subunit), and *petN* (cytochrome b_6_/f complex subunit). This coordinated upregulation suggests that IAA enhances the synthesis and assembly of photosynthetic complexes, thereby maintaining the structural integrity and functional activity of the electron transport chain. The higher Fv/Fm values in IAA-treated cultures could thus be attributed to improved PSII efficiency and more efficient electron flow from PSII to PSI, which would reduce the excitation pressure and mitigate photoinhibition. Nevertheless, the persistent concentration-dependent decrease in Fv/Fm even in the presence of IAA indicates that although IAA substantially improves photosynthetic adaptability, it does not completely abolish SMX-induced photoinhibition. This suggests that at high SMX concentrations, the toxic effects may overwhelm the protective capacity of IAA, leading to irreversible damage to PSII reaction centers or sustained inhibition of the repair cycle.

**Figure 5:**
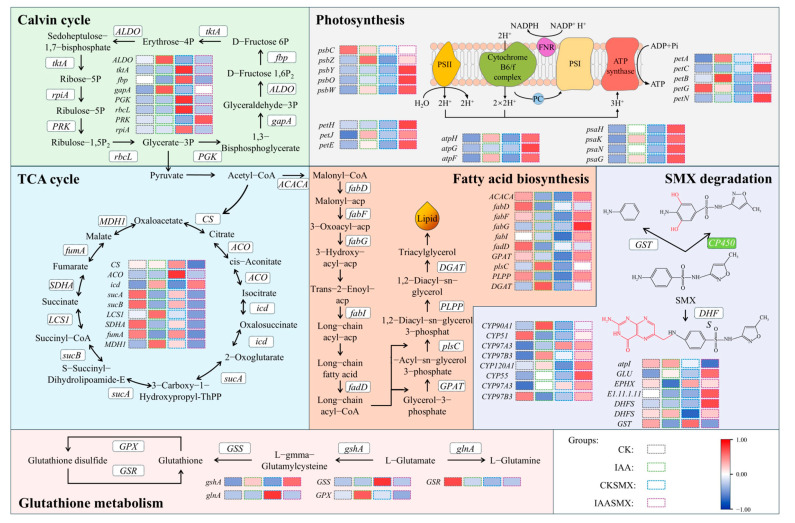
Variation of gene expression related to photosynthesis, the Calvin cycle, the TCA cycle, fatty acid biosynthesis, SMX degradation, and glutathione metabolism pathways in *C. vulgaris* from different experiments.

Regarding the antioxidant response of *C. vulgaris* under SMX stress, the lipid peroxidation cascade amplifies oxidative stress through the generation of lipid radicals, ultimately leading to the degradation of essential cellular components such as proteins and DNA [50]. Although low SMX concentrations did not significantly elevate MDA levels, the observed modulation of antioxidant enzyme activities (e.g., SOD and CAT) indicates the activation of an early oxidative stress response rather than the complete absence of oxidative effects. In contrast, higher SMX concentrations resulted in significant MDA accumulation, reflecting overt oxidative membrane damage. Furthermore, the attenuation of LDH release by IAA supplementation indicates that IAA might mitigate SMX-induced membrane disruption. Mechanistically, SMX exposure is known to induce overproduction of ROS in microalgae, primarily through disruption of photosynthetic electron transport and mitochondrial respiration [51]. Excessive ROS accumulation could trigger peroxidation of polyunsaturated fatty acids in the membrane lipids, leading to loss of membrane fluidity, increased permeability, and ultimately leakage of cytoplasmic LDH. Additionally, ROS could cause the oxidation of membrane proteins and damage to ion channels and transporters, thereby further compromising the integrity of the membrane [52]. The protective effect of IAA is therefore likely mediated by its ability to enhance antioxidant enzyme activities (e.g., SOD, CAT, and GSH-Px) (Figure 2a) and/or directly scavenge SMX by upregulating relevant functional genes of CYP450 (Figure 5), thereby reducing oxidative damage to the plasma membrane. This is consistent with the observed reduction in LDH release in IAA-supplemented cultures and supports the role of oxidative stress as a primary driver of SMX-induced membrane dysfunction.

Subsequently, the observed changes in antioxidant enzyme activities in this study provide important insights into the physiological status of *C. vulgaris* under SMX stress and the protective mechanism of IAA. In the control group without IAA supplementation, high-concentration SMX stress led to decreased activities of SOD and GSH-Px, implying that the cells sustained severe oxidative damage, which compromised their capacity to synthesize or maintain the functionality of SOD and GSH-Px proteins. Such impairment likely results in the accumulation of superoxide anions (O_2_^−^), subsequently attacking the cell membrane, proteins, and photosynthetic apparatus, which is consistent with the observed increases in LDH release and decreases in Fv/Fm [50]. Meanwhile, the sharp increase in CAT activity might represent a compensatory stress response upon H_2_O_2_ burst [53], while IAA supplementation could alert the antioxidant behavior of the microalgae. In the IAA-treated group, the enhanced SOD activity under high-concentration SMX stress demonstrates that IAA might reinforce the algal capacity to sustain or upregulate SOD expression under severe stress. This enhancement may arise from IAA-mediated upregulation of antioxidant gene transcription or protection of SOD protein stability [38]. The elevated SOD activity ensures efficient scavenging of O_2_^−^. Importantly, CAT activity in the IAA-treated group did not exhibit the stress-induced increase observed in the control group; instead, it remained stable or even slightly declined. This reflects that, under IAA regulation, the cells maintained a favorable redox homeostasis through multiple synergistic mechanisms (e.g., enhanced SOD and GSH-Px activities) [53].

In parallel with its role in oxidative defense, IAA also enhances SMX removal through both biosorption and biodegradation pathways. Biosorption refers to the adhesion of antibiotic molecules to algal cell surfaces via electrostatic attraction, hydrogen bonding, and van der Waals forces between functional groups on the cell wall and the antibiotic. The biosorption capacity of microalgae is known to be influenced by biomass concentration [54]. Moreover, IAA supplementation may stimulate the secretion of extracellular polymeric substances (EPS), which have been demonstrated to enhance antibiotic adsorption through interactions between SMX and amide groups, polysaccharides, and other functional moieties [55].

More importantly, biodegradation was inferred as the predominant mechanism for SMX removal, involving the enzymatic transformation of antibiotics into simpler metabolites within algal cells. Previous studies have shown that alterations in cellular physiological states, such as those induced by salt stress, can enhance the pollutant removal capacity of microalgae, often accompanied by changes in cell size, morphology, and gene expression [11]. Model green microalgae such as *Chlamydomonas reinhardtii* have played a central role in elucidating the molecular basis of stress adaptation, xenobiotic detoxification, and lipid metabolism. As reported by Wang et al. [56], *Chlamydomonas reinhardtii* adapts to SMX stress by modulating cellular metabolism, reinforcing oxidative stress responses, and increasing SMX adsorption as the strategy that offers generalizable insights into algal bioremediation under antibiotic pressure. Recent studies have provided genetic evidence for endogenous IAA biosynthesis and its regulatory role in algal-bacterial mutualism and stress modulation in *C. reinhardtii* [23]. These mechanistic insights offer a valuable framework for interpreting hormone-mediated responses in other chlorophyte microalgae, including *Chlorella* species. Although species-specific differences in metabolic regulation exist, the conserved auxin-associated redox and metabolic pathways suggest that the regulatory effects observed here may reflect a broadly transferable stress-adaptation strategy among green microalgae.

The biodegradation of SMX has been reported to be primarily mediated by intracellular enzymes, notably the cytochrome P450 (*CYP450*) monooxygenase family, which catalyzes the initial hydroxylation of SMX via oxygen insertion into C–H or N–H bonds [57]. Critically, it is unlikely that IAA has the capacity to degrade SMX directly; rather, it may alleviate oxidative stress and preserve the activity of these degradation enzymes, thereby indirectly and synergistically enhancing removal capacity. Thus, IAA modulates algal physiology to potentiate both biosorption and biodegradation, with a particularly marked effect under high SMX stress.

In summary, IAA enhances the tolerance of *C. vulgaris* to SMX by restoring redox homeostasis rather than merely elevating individual antioxidant enzymes. Under SMX stress, excessive ROS disrupts cellular redox balance and impairs photosynthetic and metabolic functions. IAA mediates a coordinated modulation of SOD, CAT, and GSH-Px activities, preventing ROS overaccumulation while avoiding excessive antioxidant activation. This balanced redox regulation limits membrane lipid peroxidation (MDA) and cellular leakage (LDH), preserves photosynthetic integrity, and maintains metabolic activity necessary for SMX biodegradation. Thus, IAA establishes a stable oxidative equilibrium that enables sustained stress tolerance and pollutant removal. Moreover, it is also worth considering the possible effects of various auxins (e.g., indole-3-butyric acid (IBA), 1-naphthaleneacetic acid (NAA), and 2,4-dichlorophenoxyacetic acid (2,4-D), etc.) on the physiology and biochemistry of *C. vulgaris*, which commonly exists in actual wastewater [58]. Piotrowska-Niczyporuk and Bajguz [59] have demonstrated that natural (IAA, IBA, and PAA) and synthetic (NAA) auxins elicit qualitatively similar physiological responses in *C. vulgaris*, including enhanced antioxidant enzyme activities (SOD, CAT, and APX) and suppressed lipid peroxidation and H_2_O_2_ accumulation, thereby supporting the central role of antioxidant defense modulation in auxin-mediated stress protection.

### 4.2. IAA Enhances Biomass Harvesting and Lipid Production

Microalgal biomass harvesting remains challenging, largely attributable to small cell size and the uniformly negative surface charge of algal cells [60]. Biomass yield variability further complicates harvesting efficiency under SMX stress. IAA is well established as a key regulator of algal cell division and growth metabolism [61]. Consistent with previous reports, phytohormone supplementation enhances algal growth rate and biomass accumulation, alleviates oxidative damage induced by exogenous pollutants, and improves stress tolerance [62,63,64]. The concurrent enhancement of SMX resistance and settling performance observed in IAA-treated *C. vulgaris* suggests that IAA supplementation may activate EPS-related metabolic pathways under SMX stress, leading to enhanced EPS secretion. EPS have been demonstrated to be critical for microalgal settling, functioning as a bio-flocculant that modulates cell surface properties and determines aggregate morphology and function [65]. In parallel, EPS serves as a protective barrier against environmental stress [26]. Stress conditions could induce substantial alterations in EPS yield, composition, and physicochemical properties, thereby directly affecting flocculation and sedimentation efficiency [66,67]. Microalgae typically upregulate EPS secretion under stress, wherein proteinaceous components adsorb toxins and polysaccharides form a physical barrier [68]. Moreover, the observed reduction in absolute zeta potential in IAA-treated cells promotes cell aggregation and sedimentation. Collectively, these findings highlight the pivotal role of IAA in enhancing both biomass accumulation and harvestability of microalgae under antibiotic stress.

The progressive decline in lipid productivity with increasing SMX concentration observed in this study aligns with earlier studies [41,69]. Here, IAA supplementation significantly increased both lipid content and biomass, offering an effective strategy to offset SMX-induced suppression of lipid production. Notably, certain previous studies have reported diminished lipid content upon phytohormone treatment, potentially due to enhanced linoleic acid metabolism or preferential carbon partitioning toward growth over storage lipid synthesis [34,64]. This discrepancy likely reflects species-specific variations in phytohormone responsiveness among microalgae [26].

Microalgal lipids constitute feedstocks for biofuel production, and their fatty acid profiles critically determine fuel properties [40]. A higher SFA content confers enhanced oxidative stability to biodiesel [40]. Moreover, the abundance of C16–C18 fatty acids is indicative of superior biodiesel conversion potential [70]. The IAA-induced enrichment of C16–C18 fatty acids observed in this study thus facilitates downstream biodiesel production [41,63]. Unsaturated fatty acids (UFAs) have been implicated in algal resistance to environmental stressors [71], with elevated UFA levels reflecting an enhanced oxidative stress response [34]. The IAA-mediated reduction in UFA content may therefore attenuate SMX-induced oxidative damage, modulate intracellular stress signaling, and remodel fatty acid saturation profiles to simultaneously improve stress tolerance and biodiesel feedstock quality. The core mechanism by which IAA promotes the accumulation of SFA and increases C16–C18 content in *Chlorella vulgaris* is orchestrated through the regulation of key genes involved in the fatty acid biosynthesis pathway. Specifically, IAA upregulates functional genes associated with malonyl-CoA synthesis and fatty acid chain elongation (e.g., *fabF*, *fabD*, and *fabI*), thereby directly enhancing the production of C16–C18 saturated fatty acids, which is consistent with previous findings [34]. Furthermore, studies [34] have reported that IAA could suppress the expression of desaturase genes responsible for converting SFA to unsaturated forms. This downregulation attenuates the further desaturation of monounsaturated fatty acids (e.g., C18:1) into polyunsaturated fatty acids (e.g., C18:2 and C18:3), resulting in the accumulation of newly synthesized fatty acids predominantly as saturated and monounsaturated species.

In summary, IAA enhances the harvestability of *C. vulgaris* under antibiotic stress through EPS-mediated modulation of cell surface properties and reduction of zeta potential, while concurrently promoting lipid accumulation and optimizing fatty acid composition for biofuel applications. These dual benefits position IAA supplementation as a multifaceted strategy to address key bottlenecks (e.g., biomass recovery and lipid productivity) in microalgae-based bioremediation coupled with biofuel production.

### 4.3. Genetic Insights of IAA Enhancement on SMX Tolerance, Removal Capacity, and Lipid Production

Firstly, the downregulation of photosynthesis-related genes under SMX stress likely reflects a strategic resource reallocation toward antioxidant defense, thereby minimizing ROS overgeneration and enhancing stress tolerance [72]. IAA appears to restore photosynthetic capacity by improving electron transport chain integrity and photosystem II stability [73]. This restoration not only elevates energy supply but also provides carbon skeletons and reducing power for anabolic processes such as lipid biosynthesis and stress acclimation [74]. Then, the upregulation of functional genes involved in the Calvin cycle under SMX alone suggests a compensatory enhancement of carbon fixation to sustain energy demands [75], while elevated *CS* expression indicates increased TCA cycle activity to support biosynthetic precursors [34]. The downregulation of these genes in IAA-treated cells implies that IAA alleviates metabolic pressure via alternative regulatory pathways, reducing the need for compensatory upregulation in central carbon metabolism.

Secondly, lipid biosynthesis in *C. vulgaris* serves dual functions: production of high-value compounds and stress adaptation through osmotic and oxidative defense [34]. IAA-driven upregulation of *ACAC*, *fabG*, *fabI*, and *fabF* expands the cellular acyl-CoA pool, promoting triacylglycerol (TAG) accumulation as a stress-responsive storage mechanism [73]. TAGs are the primary biodiesel precursors, and their enhanced synthesis positions IAA as a metabolic modulator capable of coupling stress defense with biofuel feedstock production [76].

Regarding antibiotic removal, the cytochrome P450 family catalyzes the initial hydroxylation of SMX [57]; their marked upregulation by IAA demonstrates direct enhancement of SMX catabolism. Concurrent induction of *GLU* and *DHFS* implicates additional detoxification routes (e.g., acetylation and formylation) in IAA-potentiated SMX degradation [77]. Furthermore, the significant upregulation of *gshA* indicates that IAA reinforced glutathione-mediated antioxidant and conjugation systems, providing complementary protection against oxidative stress [34].

Collectively, these transcriptional responses reveal coordinated metabolic reprogramming. SMX stress inhibits growth and redirects fixed carbon toward stress mitigation (Figure 1). IAA partially relieves this inhibition by restoring photosynthesis and optimizing carbon flux, thereby channeling substrates toward TAG synthesis. SMX also induces oxidative stress, generating excess NADPH; lipid biosynthesis acts as a major NADPH sink [78]. IAA alleviates oxidative damage while maintaining reductive pressure that drives lipid accumulation. Critically, IAA specifically upregulates key lipogenic genes, positioning it not merely as a mitigatory agent but as an active signal that reprograms cellular metabolism from passive defense to active resource storage [34]. While various phytohormones have been reported to enhance microalgal stress tolerance through stimulation of antioxidant defenses and metabolic activity, these effects often reflect a broader hormonal growth-promoting response [79,80]. For example, gibberellins have been shown to enhance SMX removal partly through increased carbon flux and cytochrome P450-mediated detoxification pathways [41]. In contrast, our findings suggest that IAA operates primarily through the stabilization of redox homeostasis, coordinating ROS scavenging with preservation of photosynthetic function and carbon assimilation. This redox-centered regulatory mechanism may distinguish IAA from other hormones whose effects are more directly linked to growth acceleration. Therefore, although phytohormone-induced stress mitigation represents a general phenomenon, the integrated coupling of redox homeostasis, photosynthesis, and lipid metabolism observed here indicates a mechanistic specificity of IAA in coordinating stress tolerance with metabolic reallocation.

In summary, transcriptomic evidence demonstrates that IAA orchestrates a multifaceted genetic expression that concurrently enhances photosynthetic resilience, lipid anabolism, and SMX detoxification. This integrated regulatory network enables *C. vulgaris* to transition from a stress-compensatory state to a stress-tolerant, production-oriented state, highlighting IAA as a potent metabolic engineer for coupled bioremediation and biofuel applications.

### 4.4. Potential Environmental Risks and Operational Feasibility of IAA

While research has demonstrated that IAA can serve as an effective enhancer in microalgae-based antibiotic wastewater treatment, the associated ecological risks must be carefully considered. For example, although IAA is a natural phytohormone signaling molecule, its exogenous application at high doses may disrupt the endogenous hormonal balance of non-target aquatic organisms [81]. Furthermore, the enhanced degradation of SMX by IAA-stimulated microalgae could yield various intermediate metabolites [37]. The ecotoxicity of these transformation products may be higher, lower, or unknown compared to the parent compound. If released into aquatic environments, they may exert harmful effects on aquatic life, thereby posing a potential threat to environmental ecosystems and human health [82]. Additionally, given the experimental evidence that IAA significantly promotes microalgal growth, caution is warranted regarding its use in areas prone to algal blooms. If the exogenous IAA applied during microalgae-based treatment is not fully assimilated and is discharged into receiving waters, it could stimulate excessive microalgal proliferation, potentially leading to harmful algal blooms and consequent ecological disruption.

Furthermore, IAA demonstrates considerable feasibility in terms of both cost and operational practicality. At the laboratory scale, studies have shown that adding IAA during the deceleration growth phase reduces the production costs of biomass, protein, and carbohydrates by 27%, 34%, and 75%, respectively [83]. Pilot-scale research corroborates these economic benefits, with IAA supplementation leading to a 55% reduction in biomass production cost and a 50% reduction in lipid production cost [84]. Regarding operational feasibility, Wei et al. [85] demonstrated that IAA treatment enhances the auto-flocculation efficiency of *C. vulgaris* by 93.75%, significantly simplifying and lowering the difficulty and cost associated with subsequent biomass harvesting. Therefore, although the purchase of IAA incurs an additional raw material cost, the substantial gains in productivity and the savings in downstream processing costs result in a significant net benefit. Moreover, the IAA-induced improvement in auto-flocculation efficiency directly addresses the critical scale-up bottleneck of microalgal harvesting, thereby markedly reducing the overall energy consumption and operational costs of the system.

### 4.5. Study Limitations and Future Perspectives

While this study elucidates the mechanistic role of IAA in enhancing SMX stress tolerance and lipid biosynthesis in *C. vulgaris* under controlled conditions, several limitations should be acknowledged to contextualize the findings. First, the experiments were conducted at a laboratory scale, which, while essential for precise mechanistic inquiry, does not fully capture the complexities of outdoor, large-scale cultivation systems involving environmental fluctuations and scale-up challenges. Second, the investigation focused solely on the effect of exogenous IAA. In natural and applied settings, phytohormones often function in complex networks; thus, the single-hormone approach, though clear, may not represent the integrated crosstalk that occurs in algal-bacterial consortia or under multifactorial stress. Third, to elicit measurable responses within a defined timeframe, SMX concentrations higher than typical environmental levels were employed in synthetic media. Consequently, the efficacy and dynamics of the IAA-mediated remediation observed here may differ in real wastewater streams characterized by lower, fluctuating pollutant loads and diverse microbial communities. Fourth, to ensure optimal growth conditions for *C. vulgaris* and experimental reproducibility, the use of BG11 medium is referred to the OECD 201 photoautotrophic conditions. Nitrate assimilation via nitrate reductase (NR) can produce N_2_O (a potent greenhouse gas), and NR presence in *C. vulgaris* means N_2_O generation cannot be excluded [86]. Furthermore, nitrate-based cultivation systems may pose greenhouse gas risks under alternative operational scenarios, warranting further investigation in applied studies. Finally, the metabolic shifts were inferred primarily from physiological and transcriptomic data; direct confirmation using techniques such as ^13^C metabolic flux analysis would provide more definitive evidence of carbon rerouting.

To bridge the gap between laboratory findings and applications, future research should prioritize: (i) validation in real wastewater matrices collected from different sources to assess IAA efficacy under authentic chemical and microbial complexity; (ii) investigation of algal-bacterial consortia dynamics, exploring how IAA influences interspecies interactions and overall system stability; (iii) pilot-scale validation under outdoor conditions to account for light/temperature variability and scale-up challenges; (iv) exploration of synergistic effects of IAA with other phytohormones or IAA-producing probiotic bacteria to enhance system robustness; (v) minimization of greenhouse gas emissions (e.g., N_2_O) through optimization of nitrogen sources and operational parameters; and (vi) multi-omics integration (e.g., metabolomics and proteomics) combined with flux analysis to quantitatively map the metabolic reprogramming induced by IAA under stress.

## 5. Conclusions

In conclusion, this study systematically compared the growth performance, pigment content, antioxidant system, biomass harvestability, and lipid production of the *C. vulgaris* under two distinct cultivation conditions: a control group and a group supplemented with IAA. Overall, the IAA-treated algae exhibited a superior physiological state, characterized by significantly higher biomass accumulation (with a 2.6- to 3.3-fold increase in cumulative maximum biomass), elevated photosynthetic content (a 2-fold increase in chlorophyll a under 9 mg/L SMX stress), and a more stable antioxidant system. The addition of IAA reduced the zeta potential of the algal cells, thereby enhancing their sedimentation and promoting biomass recovery. Concurrently, IAA significantly boosted both lipid yield and content (by 3.4- to 4.3-fold), with a marked increase in the proportion of C16-C18 fatty acids, which holds positive implications for subsequent biodiesel production. Furthermore, IAA effectively improved the removal efficiency of SMX by 1.14- to 2.1-fold, with biodegradation inferred as the primary removal mechanism. Transcriptomic analysis corroborated these physiological observations at the molecular level, revealing that IAA significantly upregulated key metabolic pathways, including photosynthesis, fatty acid synthesis, and SMX degradation. Collectively, this study demonstrates that functional enhancement of *C. vulgaris* via IAA supplementation establishes a sustainable technological pathway for simultaneous high-efficiency antibiotic degradation and high-value biomass production. The core mechanism lies in IAA’s coordinated activation of the algal antioxidant system and repair of photosynthetic function, which synergistically enhances the alga’s tolerance to SMX stress, its degradation efficiency, and its metabolic vitality. This integrated approach enables the concurrent achievement of efficient antibiotic removal and rapid accumulation of high-quality biodiesel feedstocks, with direct and significant implications for designing practical wastewater treatment–biofuel integrated systems. The IAA-induced enhancements collectively transferred to improved overall system performance and economic viability: efficient pollutant degradation is coupled with high biomass productivity; the facilitated harvesting lowers downstream processing energy and cost; and the elevated yield of high-quality, biodiesel-suitable lipids increases the value of the recovered bioresource.

## Figures and Tables

**Table 1:** IAA and SMX dosage in different experimental groups.

Group	IAA (mg/L)	SMX (mg/L)
1	Without IAA dosage	0
2		1
3		3
4		5
5		7
6		9
7	With IAA dosage (10 mg/L)	0
8		1
9		3
10		5
11		7
12		9

## Data Availability

The original contributions presented in this study are included in the article/Supplementary Materials. Further inquiries can be directed to the corresponding author. The raw RNA-seq data supporting this study are available in the NCBI Sequence Read Archive under the BioProject accession number PRJNA1430207.

## Supplementary Materials

The following supporting information can be downloaded at: https://www.mdpi.com/article/10.3390/microorganisms14040769/s1, Text S1: The method of solid-phase extraction and LC-MS/MS; Text S2. Analysis of antibiotics; Table S1: Instrumental analysis of LC-MS/MS; Table S2: Functional genes within the core metabolic pathways of *C. vulgaris* cells in this experiment along with distribution across different samples.

## Abbreviations

The following abbreviations are used in this manuscript:

*C. vulgaris*	*Chlorella vulgaris*
OECD	Organization for Economic Co-operation and Development
SMX	sulfamethoxazole
MDA	malondialdehyde
SOD	superoxide dismutase
LDH	lactatedehydrogenase
CAT	catalase
GSH-Px	glutathione peroxidase
OD	optical density
EPS	extracellular polymeric substances
UFA	Unsaturated fatty acids
SFA	Saturated fatty acids
LAO1	L-amino acid oxidase
TAG	triacylglycerol
GC-MS	gas chromatography-mass spectrometry
CK	control
IAA	treated with indoleacetic acid only
CKSMX	treated with SMX only
IAASMX	co-treated with indoleacetic acid and SMX
DEGs	differentially expressed genes
GO	Gene Ontology
KEGG	Kyoto Encyclopedia of Genes and Genomes
