# Supplementary material for: The Utilization of Indoleacetic Acid to Enhance the Tolerance of Microalgae to Antibiotics, Removal Capability, and Lipid Production
Source: Microorganisms. 2026 Mar 27;14(4):769. doi: 10.3390/microorganisms14040769 (PMC13118848; doi:10.3390/microorganisms14040769)
Supplement: Supplementary file 1 [file microorganisms-14-00769-s001.zip › microorganisms-4204610-supplementary.pdf]

Supplementary Materials for

The Utilization of Indoleacetic Acid to Enhance the Tolerance of Microalgae to Antibiotics, Removal Capability, and Lipid Production

Lifeng Wang [1,2], Yibo Zhang [1,2], Zhenbing Wu [1,2,3], Chenyuan Dang [1,2] and Jie Fu [1,2,*]

[1] Hubei Key Laboratory of Multi-Media Pollution Cooperative Control in Yangtze Basin, School of Environmental Science and Engineering, Huazhong University of Science and Technology, Wuhan 430074, China
[2] Green Energy Industry Research Centre (GEIRC), Huazhong University of Science and Technology, Wuhan 430074, China
[3] Hubei Key Laboratory of Purification and Application of Plant Anti-Cancer Active Ingredients, Hubei University of Education, Wuhan 430205, China
* Correspondence: jiefu@hust.edu.cn; Tel.: +86-27-87792101

Text S1. The method of solid phase extraction and LC-MS/MS

The culture medium was filtered with 0.45 μm micron nylon membrane, and diluted to 50 ml. The 30 ng of Sulfamethoxazole-13C6 as internal surrogate standard was added into sample. Then the sample solution was extracted solid phase extraction (SPE), with flow rate of 4–8 ml/min, rinsed the beakers by 10 ml ultra-pure water after samples were all passed through the cartridges, and then evacuated for one hour. The analyte was eluted with acetonitrile and dried in a 37°C water bath under a nitrogen stream. The samples of different concentration were reconstituted with different amounts of methanol. Then added different volumes of Atrazine-d5 internal standards (IS) to these four groups, and made the concentration of Atrazine-d5 in each sample is 20 ng/ml. The sample solution was then transferred to an autosampler vial (with insert) for LC/MS analysis.

Text S2. Analysis of antibiotics

Liquid chromatography tandem mass spectrometry (LC-MS/MS, Agilent 1290/6460, USA) was applied to the measurement of SMX concentrations. In the present work, the optimized method was validated, and accuracy (recovery, 68–94 %), and precision (relative standard deviation (RSD), 1.17 %–3.36 %), and sensitivity (limits of quantification (LOQ), 46.2 ng/L; limits of detection (LOD), 12.7 ng/L). Based on the requirement OECD Test Guideline, the error between nominal and actual concentration should be maintained within 20 %.

Table S1. Instrumental analysis of LC-MS/MS.

Column	Agilent ZORBAX Eclipse Plus C18 HPLC column (3 × 100 mm, 1.8 μm)		
Mobile phase	A: ultrapure water with 0.1% formic acid (v/v)<br>B: acetonitrile		
Column tem.	24 °C		
Injection volume	10 μL		
Flow rate	0.3 mL/min		
Gradient	A (%)	B (%)	
0.00 min	92.5	7.5	
1.00 min	92.5	7.5	
3.00 min	88.0	12.0	
4.50 min	80.0	20.0	
6.00 min	40.0	60.0	
9.00 min	10.0	90.0	
10.00 min	10.0	90.0	
11.00 min	92.5	7.5	
MS/MS determination	SMX	SMX -^{13}C6	Atrazine-d5
Retention time	7.06 min	7.07 min	8.58 min
Ion transitions	254.0 > 155.9<br>254.0 > 108.0	260.0 > 162.0<br>260.0 > 98.0	221.0 > 101.0<br>221.0 > 137.0<br>221.0 > 179.0
Fragmentation voltage	80 V	110 V	113 V
Collision energy	15 eV, 25 eV	15 eV, 25 eV	30 eV, 25 eV, 20 eV

Table S2. Functional genes within the core metabolic pathways of chlorella cells in this experiment were identified based on KEGG annotation, along with distribution across different samples. Core metabolism includes Photosynthesis, TCA cycle, Calvin cycle, Fatty acid biosynthesis, Glutathione metabolism and SMX degradation.

Photosynthesis

KO_id	CK	IAA	CKSMX	IAASMX	KO_id	CK	IAA	CKSMX	IAASMX
K02707	0.3767	1.44	0.15	1.4567	K02641	0.3733	0.3233	0.03	1.9833
K02704	1.2667	2.7367	1.28	1.1567	K08906	0	0.4133	0.16	0.5033
K02705	4.1867	5.5167	2.2633	3.5567	K02638	311.33	822.32	257.5067	1472.557
K02724	1.3933	4.2267	0.5867	2.8467	K02634	0.1533	0.7	0.3967	0.4833
K02709	0.25	0.9833	0.2833	1.3367	K02636	0.0733	0.1967	0.7067	2.72
K03541	0.02	0.2867	0.2133	2.3767	K02635	0.18	0.4467	0.0933	0.1
K02723	20.86	22.9867	16	56.2933	K03689	0.5267	0.98	0.5333	4.4267
K08230	0.11	0.4067	0.24	1.69	K02113	0.1333	0.66	0.4167	2.2133
K08902	0.0433	0.0867	0.0333	0.1833	K02115	1.1767	2.83	1.9633	11.7133
K08903	0.04	0.5267	0.2067	0.7433	K02109	245.4133	375.56	244	420.6433
K08901	1.7267	0.2433	1.4633	0.37	K02691	0.0633	0.45	0.0433	0.1133
K02721	423.1767	1350.777	418.9633	2039.713	K02692	0.1933	1.0033	0.33	2.3867
K02712	0.2567	0.9033	0.1333	0.6033	K02695	41.3067	107.3333	40.7367	221.8067
K03542	0.4367	1.4067	1.7467	0.34	K02698	106.93	405.1067	122.58	533.0467
K02717	0.4067	0.16	0.0533	0.6933	K02699	0.1067	0.6067	0.0167	0.3633
K08905	133.1033	418.0433	124.3233	651.5	K02701	63.16	187.2467	73.95	370.7033

K14332	1.77	12.92	8.5733	17.7233					

TCA cycle

K01647	37.8533	38.45	46.96	20.6833	K01899	1.8333	3.3233	1.6	0.6133
K00025	0.0367	0.18	0.11	0.0133	K00658	0.1633	0.0167	0.13	0.0067
K01676	4.8267	0.4	4.3367	0.15	K00164	21.82	0.6833	13.5367	0.1267
K00234	0.5633	0.0567	0.3433	0	K00031	82.2067	155.05	76.28	167.1433
K01681	0	0	2.41	0					

Calvin cycle

K01623	37.8533	38.45	46.96	20.6833	K01601	1.8333	3.3233	1.6	0.6133
K00615	0.0367	0.18	0.11	0.0133	K00927	0.1633	0.0167	0.13	0.0067
K01807	4.8267	0.4	4.3367	0.15	K00134	21.82	0.6833	13.5367	0.1267
K00855	0.5633	0.0567	0.3433	0	K03841	82.2067	155.05	76.28	167.1433

Fatty acid biosynthesis

K12405	1.02	0	1.45	0	K10258	0.73	1.9933	0.8367	0.4667
K01068	1.3333	0.0267	0.86	0.0233	K10249	0.5467	0.05	0.7	0
K10203	8.82	0.0567	7.0967	0.0167	K07513	0	0.1	0.0433	0.29
K10226	1.5667	0.2367	6.0033	0	K03921	4.5333	1.2433	7.7933	0.0333
K10703	0.04	0.1067	0.0567	0.08	K00507	7.1333	7.0133	9.4067	10.5733
K00252	0.74	0.0633	0.8367	0	K15075	0.3567	0.6033	0.0833	0.37
K10246	18.8133	0.1267	14.4033	0.1567					

**Glutathione metabolism**									
K00432	0.7367	0.0633	0.7433	0	K00797	1.87	0	0.9133	0
K00681	0	1.1233	0.16	0.2933	K23790	1.52	0.2033	2.2733	0.1067
K14262	0.42	1.08	0.7633	0.7367	K04097	18.8733	1.58	57.3633	0
K11140	4.39	3.1367	2.18	0.7567	K11188	13.6133	25.35	15.07	18.6
K01581	0.2233	7.0967	2.2967	2.6933	K21888	0.2333	0.3533	0.3133	0.7933
K01469	0.48	2.58	0.8367	3.2233	K00383	1.1933	0.09	0	0.0133
K01919	0.5967	1.65	0.6333	2.13	K00432	9.19	13.7133	9.4867	7.5567
K10808	2.86	1.4767	1.6167	1.4867	K10807	1.3567	1.9133	0.63	0.0267
K01256	0.15	0.2933	0.1633	0.3467					
**SMX degradation**									
K05917	0.78	1.69	0.94	0.4267	K15001	2.2367	0.4133	0.8733	0.06
K09837	0.26	0.1833	0.0667	0.29	K07427	0.2233	0.0467	0.7133	0.0733
K15747	11.0067	12.6733	16.4867	15.9067	K15001	0.5267	0.1233	0.48	0.0133
K09837	7.4	6.41	7.5533	6.57	K09588	0.12	0.3867	0.05	0.2367
K09837	0.3467	0.0433	0.0533	0.08	K17731	3.4333	0.6467	4.0433	0.06
K24543	0.0333	0.18	0.0467	0.1633	K07426	0.9033	0.0633	1.4067	0
K05917	4.8733	7.39	3.6367	1.55	K15001	1.0233	0.1867	1.0333	0.0267
K24543	11.0667	3.5067	5.5767	9.75	K07440	0.1733	0.0067	0.2667	0.01
K05917	4.8033	0.3833	10.6967	0.0833	K07440	1.9333	0.34	3.0067	0.1367
K24543	0.2567	3.8067	2.19	3.1867	K05917	2.22	0.7733	1.2	0.3667

K24392	25.3167	33.3133	14.18	42.0333	K15747	0.43	0.21	0.27	0
K09588	0.5467	1.87	0.2867	0.8333	K09567	3.6733	0.28	8.59	0.0933
K15001	0.4267	0.2333	1.4	0.0567	K05917	0.6	0.3	0.1833	0.21
K05917	16.9867	7.81	8.96	12.42	K15877	0.1367	0.28	0.1733	0.4733
K07426	14.8233	0.8233	18.5833	0.13	K07424	0.6167	0.1367	0.47	0.0167
K15747	0.0333	0.27	0.33	0.4					
